# Moderators of the response to deep TMS for smoking addiction

**DOI:** 10.3389/fpsyt.2022.1079138

**Published:** 2023-01-09

**Authors:** Roman Gersner, Noam Barnea-Ygael, Aron Tendler

**Affiliations:** ^1^BrainsWay, Burlington, VT, United States; ^2^Department of Life Sciences, Ben-Gurion University of the Negev, Be’er Sheva, Israel

**Keywords:** rTMS (repetitive transcranial magnetic stimulation), deep TMS, smoking cessation, moderator, predictor

## Abstract

**Introduction:**

Deep repetitive transcranial magnetic stimulation (Deep TMS™) was recently cleared by the FDA as a short-term treatment for smoking cessation. However, it is unknown which participants are more likely to benefit from the treatment.

**Methods:**

We evaluated the data from the published randomized controlled trial of 262 participants 22–70 years old that led to the FDA clearance to characterize demographic and smoking history factors that moderate Deep TMS treatment efficacy. The current analysis included 75 completers in the active TMS group and 94 completers in the sham TMS group.

**Results:**

We found that participants younger than 40 had four times the quit rate than those older than 40. Additionally, participants who quit following treatment smoked 10 years less than non-quitters. Moreover, Caucasian participants had two times the quit rate than African–American participants. Strikingly, participants with more than 12 years of education had 7 times the quit rate than participants with less education.

**Conclusion:**

Three weeks of Deep TMS has a higher smoking addiction quit rate in participants who are younger, more educated, Caucasian and with less extensive smoking history. Participants who are older, with less education and more extensive smoking history may need a longer treatment course and/or combined treatment modalities. Potential reasons may be related to the challenges of inducing neuronal modifications in those with greater physical and psychological dependence. Further investigation is warranted.

## Introduction

In the last decade, brain stimulation techniques are gradually entering clinics as a treatment option for treatment resistant brain disorders (1). Transcranial magnetic stimulation (TMS) is a safe and well-tolerated method to non-invasively stimulate the brain. Using an electromagnetic coil placed over the scalp, brief electromagnetic pulses are delivered to the underlying brain areas, to induce electrical currents in the underlying cortical tissue through induced neuronal depolarization. Repetitive TMS (rTMS) pulses applied in daily sessions induce long-term modification in mood and behavior *via* measurable structural brain changes (2). Multicenter randomized controlled trials (RCTs) demonstrated both safety and efficacy of Deep TMS coils, including the H1 Coil over lateral cortical areas for the treatment of major depressive disorder (3), the H7 Coil over medial cortical areas for obsessive-compulsive disorder (4), and the H4 Coil over the bilateral insula and prefrontal cortices for the treatment of smoking addiction (5, 6), which led to clearance of these treatments by the US Food and Drug Administration (FDA).

Brain lesions disrupting nicotine addiction map consistently to the insula or its’ connectome map (7). Which is why studies of rTMS have targeted those regions (8). In the current report we analyzed pretreatment data from the smoking RCT to characterize who is more likely to benefit from the treatment. Based on the 2020 Surgeon general smoking cessation report (9) and other reports we hypothesized that people with less education (10–13), those who are older (10, 12, 14, 15), those with greater amount and frequency of cigarette smoking (13, 14) and racial/ethnic minorities (16, 17), will have lower quit rates. Additionally, it has been shown that greater weight concerns predict a lower likelihood of both smoking cessation and reduction (18, 19), so, we examined the effect of baseline BMI on the quit rate.

## Methods

### Participants

Inclusion criteria included participants who smoke chronically (> 10 cigarettes/day for > 1 year) who met DSM5 criteria for TUD, were 22–70 years old, were motivated to quit, and had no period of abstinence > 3 months in the past year. Exclusion criteria included current treatment for smoking, use of nicotine other than through cigarettes, other active primary Axis I diagnosis or severe neurological impairment, or increased risk of seizures. A total of 262 adults met inclusion and exclusion criteria and all participants provided written informed consent. The study was conducted in the USA (12 sites) and Israel (2 sites), with active enrollment using media advertisement from August 2014 through August 2019. Please see Zangen et al. (6) for a full description of inclusion and exclusion criteria and for an elaborated methodology.

### Procedures

A central interactive web-based randomization system (IWRS) assigned a unique participant randomization code, which matched pre-programmed cards maintained at the centers and determined the nature of rTMS (Active/Sham), such that participants, operators and raters were blinded to the treatment condition. The blinding assessment (in which participants were required to guess whether they received active or sham treatment) indicated that the majority of participants in each group did not know which treatment they received (6). Following collection of demographic and smoking history data, randomization, and selection of a target quit date within the first 2 weeks of treatment (“grace period”), daily Deep TMS (active or sham) was applied for 3 weeks (five sessions/week), followed by once a week Deep TMS for three more weeks (i.e., a total of 18 Deep TMS™ treatment sessions over 6 weeks). The participants provided daily smoking diaries and once a week urine samples for assessment of cotinine levels. At each visit, the number of cigarettes smoked was recorded through the Nicotine Use Inventory, and adverse events were monitored (6).

### TMS

Treatment was delivered after a brief smoking provocation to induce cravings, using a Magstim Rapid^2^ TMS stimulator (Magstim, UK) equipped with the H4 Coil (BrainsWay, Israel) designed to stimulate the bilateral insular and prefrontal cortices (6). A sham coil is encased in the same helmet and induces acoustic and scalp sensations similar to those induced by the active coil, but without electromagnetic penetration into the brain and without neural activation (5). During each session, 60 rTMS trains of 30 pulses were applied at 10 Hz (3 s each train) with 15 s intertrain interval. A 2-min motivational talk based on the booklet “Clearing the Air” (20) and supporting the decision to quit, was read to each participant followed each treatment.

### Outcome measures

In the original study, the primary outcome measure was the 4-week continuous quit rate (CQR) until week 18 among participants composing the intent-to-treat efficacy set (i.e., the percentage of quitters among all randomized participants who met eligibility criteria and had at least one post-baseline assessment). Secondary endpoints included the CQR until Week 18 in the completer analysis set (i.e., the percentage of quitters among all randomized participants who received all 18 treatment sessions) and the CQR until Week 6.

### PICO

P: participants who smoke chronically (> 10 cigarettes/day for > 1 year) who met DSM5 criteria for TUD, were 22–70 years old, were motivated to quit, and had no period of abstinence > 3 months in the past year.

I: deep repetitive transcranial magnetic stimulation: 60 rTMS trains of 30 pulses were applied at 10 Hz (3 s each train) with 15 s intertrain interval.

C: sham repetitive transcranial magnetic stimulation: induces acoustic and scalp sensations similar to those induced by the active coil, but without electromagnetic penetration into the brain and without neural activation.

O: 4 week continuous abstinence from smoking.

### Data analysis

As the purpose of the current study was to assess the factors moderating response to a full treatment course of 18 Deep TMS sessions, we analyzed the results of the completers’ data set from the published randomized controlled trial (6) (*N* = 75 in active TMS group including 21 quitters and 54 non-quitters; and *N* = 94 in sham TMS group including 11 quitters and 83 non-quitters) at the end of treatment (Week 6) and at the end of follow-up (Week 18) unless mentioned otherwise (see Race below). The two-factorial parametric data were analyzed with the two-way ANOVA, with treatment condition (active vs. sham) and CQR (quitter vs. non-quitter) being between-subject factors. Significant main effects were followed by two-tailed *t*-test analysis for independent samples of treatment groups within each group. The two-factorial proportions data were analyzed with the Cochran–Mantel–Haenszel test, with treatment condition (active vs. sham) and age (older than 40 or younger than 40 years old), race (Caucasian or African–American) or education level (12 or less or more than 12 years of education) being between subjects factors, followed-up by *Z*-test to compare between two proportions. A significance level (α) of *p* < 0.05 was set for all statistical analyses. All data are presented as either means ± SEM or proportions.

## Results

### Age

**FIGURE 1:**
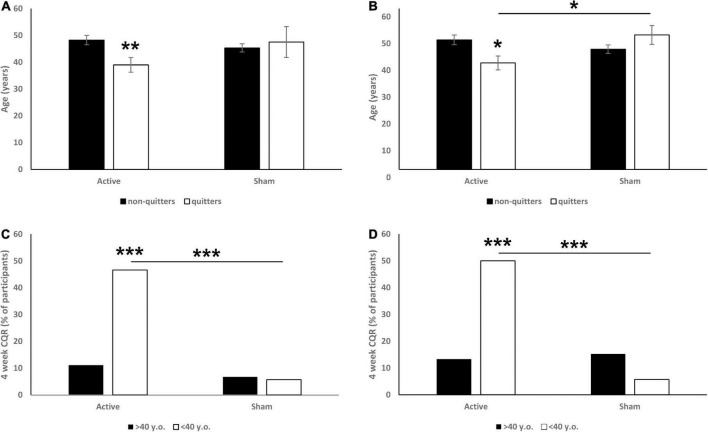
Effects of participant’s age (**A:** 6 weeks CQR data, **B:** 18 weeks CQR data) and being older or younger than 40 years old (**C:** 6 weeks CQR data, **D:** 18 weeks CQR data) on CQR. On panels **(A,B)** data are presented as mean ± SEM. On panels **(C,D)** data are presented as % of participants. *p < 0.05, **p < 0.01, ***p < 0.001.

Two-way ANOVA (with treatment and CQR as the main factors) at 6 weeks CQR data showed non-significant interaction tendency [*F*(1,165) = 2.8, *p* = 0.0948; Figure 1A], and further analysis revealed that quitters in the active arm were significantly younger (*p* = 0.008). This interaction at 18 weeks was found to be significant [*F*(1,165) = 6.2, *p* = 0.013; Figure 1B], and *post hoc* analysis revealed again that quitters in the active arm were significantly younger (*p* = 0.011). Moreover, at 18 weeks the quitters in active arm were significantly younger than quitters in sham arm (*p* = 0.025).

For further investigation we observed the efficacy in 10-year bins and noticed a drop in quit rate in participants older than 40. Specifically, we revealed a significant main effect of age (< 40 vs. > 40 years old) on CQR at both week 6 (χ^2^ = 11.73, *p* < 0.001; Figure 1C) and week 18 (χ^2^ = 6.99, *p* = 0.008; Figure 1D). *Post hoc* analysis revealed that in the 6 weeks data, younger participants from the active group had a significantly higher CQR than both older participants from the active group (*p* < 0.001) and younger participants from the sham group (*p* < 0.001). In the 18 weeks data we found similar effects: younger participants from the active group had a significantly higher CQR than both older participants from the active group (*p* < 0.001) and younger participants from the sham group (*p* < 0.001). No differences between older and younger participants were observed in the sham groups.

### Education

**FIGURE 2:**
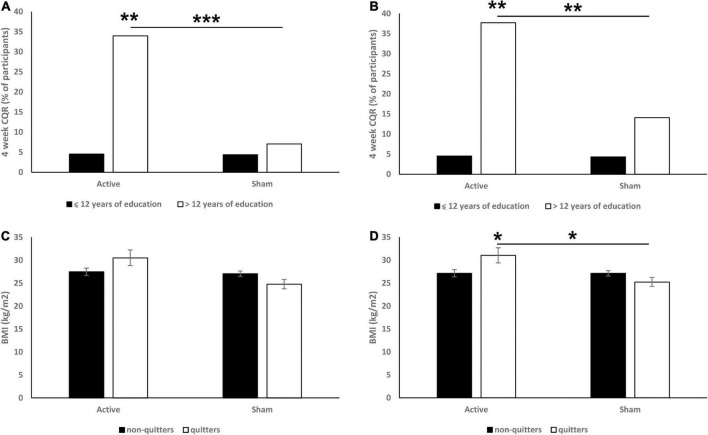
Effects of education (**A:** 6 weeks CQR data, **B:** 18 weeks CQR data) and BMI (**C:** 6 weeks CQR data, **D:** 18 weeks CQR data) on CQR. On panels **(A,B)** data are presented as % of participants. On panels **(C,D)** data are presented as mean ± SEM. *p < 0.05, **p < 0.01, ***p < 0.001.

The numbers of years of education (≤ 12 vs. > 12 years) were found to be a significant moderator of CQR at both 6 (χ^2^ = 13.13, *p* < 0.001; Figure 2A) and 18 (χ^2^ = 8.5, *p* = 0.004; Figure 2B) weeks. Specifically, we found that in the 6 weeks data, more educated participants from the active group had a significantly higher CQR than both less educated participants from the active group (*p* = 0.008) and more educated participants from the sham group (*p* < 0.001). In the 18 weeks data we found similar effects: more educated participants from the active group had a significantly higher CQR than both less educated participants from the active group (*p* = 0.003) and more educated participants from the sham group (*p* = 0.002). Years of education had no effect on CQR within the sham group.

### BMI

In the 6 weeks CQR data, analysis revealed significant main effect of treatment [*F*(1,164) = 4.5, *p* = 0.036; Figure 2C]. *Post hoc* analysis revealed that quitters in the active group had a non-significantly higher BMI than non-quitters (*p* = 0.074).

In the 18 weeks CQR data, analysis revealed a significant main effect of treatment (*F* = 6.0, *p* = 0.016; Figure 2D) and significant interaction between treatment and CQR [*F*(1,164) = 5.9, *p* = 0.016]. *Post hoc* analysis revealed that quitters in the active group had a significantly higher BMI than both non-quitters (*p* = 0.018) and quitters in the sham group (*p* = 0.022).

BMI had no effect on CQR of the sham group in both 6 and 18 weeks data, and no differences in the CQR between underweight (BMI < 18.5), healthy (18.5 < BMI < 25), overweight (25 < 29.9) or obese (> 30) participants were found either.

### Race

**FIGURE 3:**
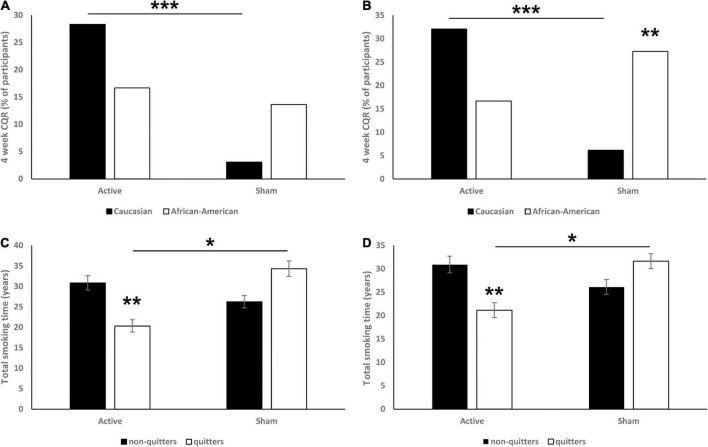
Effects of race (**A:** 6 weeks CQR data, **B:** 18 weeks CQR data) and total years of smoking (**C:** 6 weeks CQR data, **D:** 18 weeks CQR data) on CQR. On panels **(A,B)** data are presented as % of participants. On panels **(C,D)** data are presented as mean ± SEM. *p < 0.05, **p < 0.01, ***p < 0.001.

Among 75 completers in active TMS group, 53 self-identified as Caucasian, 18 as African–Americans, 2 as Hispanic, and 2 as Other. Similarly, among 94 completers in sham TMS group, 65 self-identified as Caucasian, 22 as African–American, 3 as Hispanic, and 5 as other (including one participant that self-identified as bi-racial: African–American and Other). The sample size of participants self-identified as Hispanic and Other was not sufficient and thus was not included in the following analysis. The race was found to be a significant moderator of CQR at both 6 (χ^2^ = 11.93, *p* < 0.001; Figure 3A) and 18 (χ^2^ = 6.99, *p* = 0.008; Figure 3B) weeks. Specifically, we found that in the 6 weeks data, Caucasian but not African–American active TMS participants had a significantly higher CQR than sham participants (*p* < 0.001). Across the sham participants, African–Americans had a non-significant tendency to have higher CQR than Caucasian (*p* = 0.066). In the 18 weeks data we found similar effects: Caucasian but not African–American participants in the active group had a significantly higher CQR than sham participants (*p* < 0.001). And across the sham participants, African–Americans had a significantly higher CQR than Caucasian (*p* = 0.007).

### Total years of smoking

In the 6 weeks CQR data, analysis revealed a significant interaction [*F*(1,165) = 7.48, *p* = 0.007; Figure 3C], and *post hoc* analysis revealed that quitters in active group had significantly fewer total years of smoking than both non-quitters (*p* = 0.002) and quitters from the sham group (*p* = 0.029).

In the 18 weeks CQR data, analysis revealed a significant interaction [*F*(1,165) = 7.37, *p* = 0.007; Figure 3D], *post hoc* analysis revealed that quitters in the active group have significantly less total years of smoking than both non-quitters (*p* = 0.004) and quitters in sham group (*p* = 0.034). Total years of smoking had no effect on CQR in sham groups.

## Discussion

The original studies reported that Deep TMS with the H4 Coil over the bilateral insula and prefrontal cortices is an effective treatment for smoking addiction (5, 6). Numerous earlier studies have focused on modulating brain regions associated with addictions such as dorsolateral prefrontal and insular cortices with different TMS protocols and suggested the mechanism is largely through reduction of cravings and increase in inhibitory control (8). In general, therapeutic interventions with transcranial magnetic stimulation are state-dependent. For example, craving induction using smoking provocation is beneficial for TMS outcomes (21). Similarly, it is feasible that participants that are less likely to respond to other smoking cessation methods (for example, from specific demographics) can be less likely to respond to TMS.

The current study examined whether previously identified demographic and smoking history factors predictive of smoking cessation also predict the beneficial effect of Deep TMS.

Indeed, we found that younger age, a relatively shorter history of smoking, higher level of education and Caucasian race are associated with increased rate of TMS mediated successful cessation.

Mechanistically, Deep TMS for addiction would require induction of long term potentiation for learning to occur (22, 23). Cortical synaptic plasticity is negatively correlated with increasing age, and while education cannot prevent neuropathology it can mitigate the severity of the symptoms expressed by it (24). Additionally, age is a factor with pronounced cessation disparities where both past-year quit attempts and recent successful cessation decrease as adult cigarette smoker’s age increase (9). Moreover, nicotine dependence—a significant barrier to smoking cessation—in people younger than 45 was found to be lower than in people 45–64 years old (25).

Participants who did not complete high school (< 12 years) had significantly lower quit ratios compared with participants with higher levels of education (undergraduate or graduate degree) (9). Moreover, in nicotine replacement therapy education was also found to predict compliance with this treatment and smoking abstinence at end of treatment (26). Disparities in level of educational attainment are closely correlated with income, poverty, overall socioeconomic status, status of health insurance and geographic location.

Cigarette exposure in pack-years, cigarette dependence and the urge to smoke were shown to negatively correlate with thickness in right insula (27). It is possible that Deep TMS changes the thickness of the insula as has been previously demonstrated with traditional TMS in depression (2).

While there was no change in BMI from baseline to endpoint in both active and sham groups (6), a positive correlation between BMI and smoking quit rate was observed only in the active TMS group. Since the insular cortex is involved in both smoking and food addiction, TMS with the H4 may address both problems (28).

According to Surgeon General report (9), there are disparities in smoking cessation between racial/ethnic minorities and the general population. For example, Black adults who smoke have significantly lower quit ratio than White adults who smoke (9, 16). It is hypothesized to reflect on disparities in socioeconomic status, and due to predatory marketing by the tobacco industry in geographic areas with large number of Black residents (29, 30). In the varenicline study lower quit rates among Blacks were correlated with lack of homeownership, lower income, and greater neighborhood problems (17). Another study also found that Blacks who smoke were less responsive to varenicline, bupropion and NRT than Whites (16). The authors also suggested that understanding of socioeconomic variables may improve outcomes for Blacks who smoke. Further, in a study which factored in financial strain and educational attainment, no difference in smoking cessation between African–Americans and Whites was observed (31). The observed higher placebo quit rate in African–American at 18 weeks may be due to racial disparities in placebo effects (32, 33) or due to small N.

The main limitation of this study is the modest sample size, which restricted more elaborate factorial analysis. However, following FDA clearance and the initiation of the treatment in the clinics, it is expected that collection of real-world data will allow more reliable analysis. The factors we have found to moderate the quit rate following TMS have been previously reported (9) to moderate existing treatments.

It is possible that participants with more negative predictors may need higher doses of stimulation or add-on of pharmacotherapy or behavioral therapy to achieve abstinence. For example, in depression—the most studied TMS indication–prolonged treatment courses and higher pulse numbers result in higher response rates (34–37). These findings warrant further validation, and these treatment recommendations should be considered if any subject does not quit smoking by the target quit date of 2 weeks of daily deep TMS.

## Data availability statement

The raw data supporting the conclusions of this article will be made available by the authors, without undue reservation.

## Ethics statement

The studies involving human participants were reviewed and approved by the Sterling Commercial Institutional Review Board, as well as local institutional review boards, and registered at www.clinicaltrials.gov (NCT02126124). The patients/participants provided their written informed consent to participate in this study.

## Author contributions

AT designed the study. RG performed the statistical analysis. All authors contributed to manuscript writing, revision, and approved the submitted version.
